# Curious Fatality Which Pursues the First-Born Sons of the Crockett Family

**Published:** 1888-11

**Authors:** 


Curious Fatality Which Pursues the First-Born Sons of the Crockett Family.—There is a certain family in Boston which suffers from a mysterious hereditary curse of the sort one reads about in hair-elevating stories of the supernatural. The first born son to every daughter bleeds to death. The story as to the manner in which the affliction originated cannot, of course, be vouched for, says the Boston correspondent of the St. Louis *Globe-Democrat.* It has to do with a wicked great-great-grandmother, who murdered a rich uncle by opening one of his veins with a poinard, in order to get possession of the old gentleman's vast wealth. The ghost of the deceased subsequently appeared to the unscrupulous niece and announced in hollow tones, appropriate to the tomb, the dismal prediction that for all time thence-forth the eldest male child of every girl in her family should die by bleeding to death.

So much for the explanatory tradition. The fact is that for many generations the Crockett family of Charlestown—which is a part of Boston—and vicinity has been pursued by a hemorrhagic Nemesis. The first son born to every daughter, even to remote cousins, invariably bleeds to death. All other members of the Crockett tribe are exempt from the mysterious trouble. But the method of this inherited curse is so well known that each female Crockett is prepared, on the arrival of her initial boy, for the experience that is bound to ensue. The first little cut, or even scratch, the infant experiences is the signal for a panic. Bandages are applied as quickly as possible, and the wound is treated with a preparation of iron in the form of a powder. It is a narrow squeak in such cases always, but there is a good chance of recovery within eight days if the thing is taken in time. At the end of that period the patient either gets well or dies from loss of blood very suddenly. For such is the manner of the bleeder's complaint. He is sure to be attacked in precisely the same way every time during his after life that his skin is seriously abraided. On occasions of the sort he must adopt immediate measures remedial or die. By exercising the most extraordinary precautions he may reach a comfortable age, but sooner or later he is sure to perish by an untoward accident, causing a flow of blood which no physician's art can stop. So far not a single one of the destined victims has escaped the penalty. The oldest one now living is a Mr. Surratt, of Melrose, who has been accustomed, when he wanted a tooth pulled, to revise his will, visit his relatives and bid them good-by, as though it were likely to be forever. Life is an extra hazardous task when you are a bleeder. Thus it happens that eldest sons of the Crockett family, direct and collateral on the maternal side, are found to be engaged in grave digging and and other harmless avocations which are not likely to occasion incidental hurts.

A curious story is it not? And yet the writer begs to offer his personal assurance that it is true in every respect.

